# Discussing methodological gaps in psychosocial intervention research for dementia: an opinion article from the INTERDEM Methodology Taskforce guided by the MRC framework

**DOI:** 10.3389/frdem.2024.1458023

**Published:** 2024-09-26

**Authors:** Sara Laureen Bartels, Nathan Stephens, Federica D'Andrea, Melanie Handley, Marine Markaryan, Andrea Nakakawa Bernal, Lieve Van den Block, Simone R. de Bruin, Karen Windle, Martina Roes, Niels Janssen, Hannah Christie, Lesley Garcia, Gwen Teesing, Esme Moniz-Cook, Maud Graff

**Affiliations:** ^1^Department of Psychiatry and Neuropsychology and Alzheimer Centrum Limburg, Mental Health and Neuroscience Research Institute, Maastricht University, Maastricht, Netherlands; ^2^The Association for Dementia Studies, School of Allied Health and Community, University of Worcester, Worcester, United Kingdom; ^3^The Geller Institute of Ageing and Memory, School of Medicine and Biosciences, University of West London, London, United Kingdom; ^4^Centre for Research in Public Health and Community Care, University of Hertfordshire, Hatfield, United Kingdom; ^5^Design Department, Politecnico di Milano, Milan, Italy; ^6^VUB-UGent End-of-Life Care Research Group, Department of Family Medicine and Chronic Care, Vrije Universiteit Brussel, Brussels, Belgium; ^7^Research Group Living Well with Dementia, Division Health and Social Care, Windesheim University of Applied Sciences, Zwolle, Netherlands; ^8^Centre for Applied Dementia Studies, University of Bradford, Bradford, United Kingdom; ^9^German Center for Neurodegenerative Diseases (DZNE), Witten, Germany; ^10^Faculty of Health, Department of Nursing Science, University of Witten/Herdecke, Witten, Germany; ^11^School of Population Health, Royal College of Surgeons in Ireland, Dublin, Ireland; ^12^School of Sociology and Social Policy, University of Nottingham, Nottingham, United Kingdom; ^13^Alzheimer Nederland, Amersfoort, Netherlands; ^14^Faculty of Health Sciences, University of Hull, Hull, United Kingdom; ^15^Department of Rehabilitation, Radboudumc, Radboud University Medical Centre Nijmegen, Nijmegen, Netherlands; ^16^Department of IQ Health, Radboudumc Alzheimer Center, Radboud University Medical Center Nijmegen, Nijmegen, Netherlands

**Keywords:** dementia care, psychosocial intervention, support, methodology, public involvement, theory-driven, context, complexity

## Introduction

“*Whilst research on psychosocial interventions in […] dementia is already showing signs of increased rigor and robustness […], there is a need to allow a variety of types and sources of evidence to influence practice, and not simply be driven by results from randomized controlled trials*” (Woods, 2003, p. 6).

This statement is over 20 years old. Yet, it remains pertinent today as dementia research still shows an over-reliance on Randomized Controlled Trials (RCTs) for testing intervention efficacy within “ideal world” or optimum conditions (Hui et al., 2021; Oyebode and Parveen, 2019). Furthermore, over 20 years ago, a hierarchical framework for ranking intervention evidence noted that the human subjective experiences of the recipient can be devalued, unless appropriate research designs are used (Evans, 2003). Despite increasing research commitment to involve people living with dementia and unpaid carers, meaningful involvement often remains superficial in many studies (Miah et al., 2019; Kirby et al., 2024). Consequently, there is a risk of research waste due to an “implementation error” where costly and time-consuming outcome evaluations including RCTs may (i) not demonstrate effectiveness but interventions themselves reported positive effect on peoples' experiences; (ii) demonstrate effectiveness but are unfeasible, unacceptable, ineffective in practice or viable only under limited circumstances (Vernooij-Dassen and Moniz-Cook, 2014). In contrast, diverse forms of evidence through the appropriate use of approaches to develop, implement, and evaluate interventions lead to more efficient, practical, and impactful research and practice (Skivington et al., 2021). Based on observations in the literature and the author's scientific views, this article draws attention to three methodological concerns: (1) people living with dementia, unpaid carers, and other stakeholders are not always *meaningfully involved*, (2) many current methods are not ideal in understanding *what works for whom, how, and why* and, (3) key features of *context and intervention complexity* are sometimes neglected.

Psychosocial interventions in dementia are considered as complex because of the intervention characteristics as well as how these characteristics interact with the inner and outer intervention context, as also described by the Medical Research Council (MRC) framework (Skivington et al., 2024). Characteristics of the intervention include, for instance, number and flexibility of components, the range of target behaviors, expertise, skills, and attitudes of health and social care professionals required, as well as people living with dementia and unpaid carers expected to receive the intervention. The context can refer to the setting in which the intervention is intended to be used, such as the country, to its policies and culture, and to the person's living situation (e.g., home-based, dementia day care, hospital, care home). The interaction between interventions and contexts is of relevance as this link is part of the mechanism of change, where causality between the intervention characteristics and outcomes can be determined (Skivington et al., 2021). Understanding causality is important so that appropriate evidence can be developed on outcomes at multiple levels [e.g., individual, service, and implementation (Proctor et al., 2011, 2023: Damschroder et al., 2022; McDermott et al., 2019; Moniz-Cook et al., 2011)]. Various frameworks can be used to develop, implement, and evaluate complex interventions (e.g., Damschroder et al., 2022; Bartholomew et al., 1998; Guise et al., 2017). The updated UK MRC aims to “*help researchers […] to design and conduct research with a diversity of perspectives and appropriate choice of methods”* (Skivington et al., 2021, p. 1). It has been cited over 5,000 times (Status: WoS May 2024), where at least 300 are connected to “dementia”. Therefore, it appears timely to reflect on its application in psychosocial dementia research.

The MRC framework outlines six core elements (i.e., consider context; develop, refine, (re)test program theory; engage stakeholders; identify key uncertainties; refine intervention; economic considerations) interacting with four phases (i.e., develop/identify intervention; feasibility; evaluation; implementation) (see Figure via link). We welcome Skivington et al. (2021, p. 1) acknowledgment that trade-offs exist for researchers between answering “*questions that are useful to decision makers rather than those that can be answered with greater certainty*”. For example, RCTs can provide evidence on the effectiveness of psychosocial interventions in dementia (Aguirre et al., 2013) but literature in medical and social sciences may overestimate the accuracy of aggregated statistical estimates (Fisher et al., 2018). The issue is also linked to the “*overconfident belief in replicability*” of statistically significant effects (Vasishth et al., 2018) and a limited generalizability from the group to the individual level (Molenaar, 2004). Unraveling intra- and inter-individual differences is especially important given the substantial heterogeneity in dementia manifestations. Although promising approaches, such as item response theory (Murray et al., 2021) or single-case experimental designs (e.g., Lagerlund et al., 2022; Yorozuya et al., 2022), have emerged to address these short-comings of RCTs, the aspect listed above are rarely considered in interpretation of psychosocial data.

Moreover, the MRC framework documents the need to consider intervention context (e.g., circumstances surrounding the intervention's development, evaluation, and/or implementation) and complexity (e.g., emergent costs and effects, multiple and interacting components and systems). These features of psychosocial dementia interventions are not always considered (Christie et al., 2018). Often, limited attention is paid to the underlying mechanisms for *how* and *why interventions work or not*, thereby reinforcing reductionist approaches of merely reporting *what* changed (Moore et al., 2019).

Overall, the MRC framework emphasizes the importance of developing, evaluating, and implementing interventions based on theory (e.g., implementation science), practice knowledge (e.g., what works or not), and lived experience involvement (e.g., preferences, values, co-approaches) (Skivington et al., 2021, 2024). In some research studies, novel methodological approaches are emerging that better acknowledge real-world contexts and recognize the importance of involving people living with dementia, unpaid carers, and other stakeholders (Phillipson and Hammond, 2018). The MRC framework has scope to guide approaches and advance psychosocial dementia research. However, it is currently unclear which designs and methodologies frequently used in psychosocial dementia research address which questions, core elements, or relate to particular phases.

In this opinion paper, we discuss methodological gaps in psychosocial intervention research for dementia as identified by members of the Methodology Taskforce of INTERDEM. We reflect on and outline approaches that align with several of the MRC framework's core elements useful for research questions related to the development, evaluation, and implementation of psychosocial interventions in dementia. Specifically, we focus on stakeholder-informed and co-approaches with people living with dementia and unpaid carers, as well as theory-driven evaluation. The overarching aim of this opinion article is to stimulate a debate and to promote best research practice in the field.

## Stakeholder-informed and co-approaches in psychosocial dementia research

All phases of the MRC framework recognize stakeholder engagement as a core element (Skivington et al., 2021). Stakeholders are defined as: individuals, groups of individuals, and organizations who affect intervention development, implementation, or evaluation (Social Value International, 2019). Within dementia research, key stakeholders include people living with dementia (defined as Public Involvement by Alzheimer Europe), unpaid carers, health and care professionals, insurers/commissioners, and decision/policy-makers.

Conducting complex interventions research alongside or with people living with dementia is essential (Gove et al., 2018), especially due to the multifaceted nature of the condition (Warran et al., 2023). Ensuring wider representation, including under-represented groups (Low et al., 2019; Vyas et al., 2018), and achieving “true” or meaningful engagement remains a challenge (Roberts et al., 2020). Empowering people living with dementia and unpair carers to participate actively in decision-making processes requires specific considerations to minimize power imbalances and avoid tokenism (Swarbrick et al., 2019; Marjanovic et al., 2015). While the MRC framework highlights the importance of stakeholder engagement, to the authors knowledge, designs and methodologies that can specifically engage and empower people living with dementia and unpaid carers are not yet utilized optimally, also neglecting underrepresented populations (e.g., ethnic minorities, immigrants, socio-economically disadvantages individuals). This issue may also be due to researchers finding it challenging to reach these populations and/or to engage people living with dementia in a meaningful way.

Participatory research, defined as an approach where researchers work in partnership with people living with dementia and unpaid carers throughout the research process, is slowly increasing in the field (Reyes et al., 2023). In practice, participatory research ranges from stakeholder involvement in an advisory role, such as reviewing research proposals, to collaborative co-approaches where power and responsibility are shared (Farr, 2018; Moll et al., 2020). Co-production, co-design, and co-creation are often used interchangeably due to limited consensus on definitions of co-approaches (Cowdell et al., 2022; Grindell et al., 2022). The MRC framework suggests that early stakeholder involvement can contribute to identifying and prioritizing ideas for research to answer “real world” questions, defining topics, gaining insight into problems, and optimizing study design/evaluation and implementation (O'Cathain et al., 2019; Skivington et al., 2021). Nonetheless, active involvement of people living with dementia and unpaid carers in designing, planning, and dissemination may be rarer due to stigmatizing narratives (Cowdell et al., 2022), top-down research, policy prioritization of epidemiological perspectives, and methodologies focusing on effectiveness, generalizability, and replicability (Warran et al., 2023). It is therefore crucial to emphasize the value of different types of data and equal collaboration with people living with dementia and unpaid carers “*to identify what ways of knowing are important*” (Warran et al., 2023, p. 5).

The most used co-approach methods with people living with dementia, unpaid carers, and stakeholders appear to be interviews or focus groups (Cowdell et al., 2022), often involving family or professional caregivers which can hinder fully capturing the voices of people living with dementia due to gate keeping (Novek and Wilkinson, 2019). Additionally, these methods usually rely on abstraction, recall, and verbal communication, which may be difficult for some people (Phillipson and Hammond, 2018). In response to these limitations, novel methods have been used (Campbell et al., 2023; Hogger et al., 2023), including visual (Chen et al., 2022), creative methods (Murphy and Oliver, 2013; Phillipson and Hammond, 2018), and sensory techniques (Buse and Twigg, 2016; Fleetwood-Smith et al., 2022) also capturing non-verbal communication. In the CONNECT study, experience-based co-design (Bate and Robert, 2006) and visual methods were used to develop an intervention that facilitates person-centered approaches to “constant observation”, a model of care allocating staff for one-to-one support or close supervision of a small group of patients in hospital. Informed by literature (Handley et al., 2023) and mapping of the practices in three hospitals, vignettes and visual illustrations in the form of storyboards represented common, reoccurring scenarios of the delivery and experience of constant observation. The “touchpoints” depicted in the vignettes and storyboards enabled people living with dementia, unpaid, and carers to react to and empathize with situations, directly influencing priorities, values, appearance, and ways to use the intervention. Similarly, in the HOMEDEM network, several projects use participatory, user-centered design, and co-design approaches to support home-based people living with dementia and unpaid carers, including iterative procedures where feedback from people targeted by an intervention is integrated repeatedly, thus, increasing the likelihood of success (Lord et al., 2022). HOMEDEM offers early-career researchers interdisciplinary training including secondments to industry partners and combines methodological knowledge of design researchers with expertise in psychology, healthcare sciences, and health economics.

These examples demonstrate the value of co-designing with diverse stakeholders, using novel approaches. Engaging co-designers at an emotional level, integrating creative materials, collaborating across disciplines, and employing iterative procedures facilitates shared understanding. Thus, people living with dementia, unpaid carers, and other stakeholders are placed at the heart of the design and research process.

## Theory-driven evaluation approaches in psychosocial dementia research

Evaluation of psychosocial interventions varies depending on the research question, targeting implementation (van Mierlo et al., 2018), effectiveness/cost-effectiveness (Brooker et al., 2018; Henderson et al., 2021), involvement (Buckner et al., 2022), sustainability (Morton et al., 2024), and scalability (Knapp et al., 2022). While evaluative studies should focus on the most proximal research question [World Health Organization (WHO, 2009)], controlled trials dominate, quantifying the effectiveness of an intervention based on “clinically meaningful” results (i.e., significance and/or effects sizes) (Skivington et al., 2021). Psychosocial dementia research is no exception (Chow et al., 2021; Teahan et al., 2020). In many ways, striving for clinical effectiveness has little moral and methodological compass as firstly, outcomes measured may not be relevant to people living with dementia and unpaid carers (Harding et al., 2019); secondly, research methods do not always detect change accurately due to power issues (Stoner et al., 2019); thirdly, effect sizes may lack comparability as results can be “*seriously inflated*”; and finally, longitudinal pragmatic RCTs are often unpracticable (Schäfer and Schwarz, 2019). Therefore, few studies can replicate effectiveness (Aarts et al., 2015) or clinically meaningful outcomes (Schulz et al., 2002), when people living with dementia or unpaid carers may experience meaningful change. Expectations of funding bodies, decision makers, and researchers regarding which evaluation approaches and evidence are appropriate have started to shift recently. Notably, questions of context and complexity are fundamental to questions of efficacy and effectiveness, for which theory-driven approaches are widely advocated (Chen, 2012; Crane et al., 2019; De Silva et al., 2014). The MRC framework (Skivington et al., 2021) could therefore signal change for the evaluation of psychosocial dementia interventions.

Theory-driven evaluation is an umbrella term for various approaches including Programme Theory (Chen, 2012), Theory of Change (De Silva et al., 2014), and realist evaluation (Pawson and Tilley, 1997). These evaluations focus on *how and why* interventions work (or not) by investigating underlying theory of change, and/or mechanisms that produce outcomes in specific contexts (Funnell and Rogers, 2011). Grounding the evaluation of psychosocial interventions in a theoretical framework that can be refined supports intervention effectiveness, sustainability, and scalability (De Silva et al., 2014) and is starting to gain traction in the field of dementia care [e.g., using Theory of Change to guide the development and evaluation of a whole-setting nursing home intervention (Gilissen et al., 2018, 2019)]. Theory-driven approaches involve stakeholders to uncover and include meaningful outcomes (Øksnebjerg et al., 2018), and open the “black box” of interventions by identifying interactive components within multi-level contexts/systems leading to change (De Silva et al., 2014; Gilissen et al., 2018). For example, realist evaluation questions “*what works, for whom, under what circumstances and how”* to generate context-mechanism-outcome configurations (CMOs) (Pawson and Tilley, 1997). As such, a realist-informed process evaluation refined a theory of collaborative improvement with diverse stakeholders to explore and quantify implementation (e.g., fidelity), process (e.g., changes in practice), and individual outcomes (e.g., knowledge) (de la Perrelle et al., 2021). Another example is the realist rapid review and realist multiple case study design as part of the MENTALITY project which were used to define underlying mechanisms for successful dementia friendly communities and initiatives (Thijssen et al., 2022, 2023).

Despite burgeoning use of realist evaluation, it is not without its criticisms. Interpreting context when forming CMOs is not straightforward. What defines a context in one example may be used as a mechanism in another, and vice versa (Shaw et al., 2018). Those using RE should be aware of and accommodate for the instability of context in the design (Greenhalgh and Manzano, 2022). For instance, realist evaluation and Soft Systems Methodology was applied to evaluate the sustainability of Meeting Centers in rural UK areas (Morton et al., 2024). Combining these approaches appears to be an effective way to model complexity, leading to a transparent programme theory (Dalkin et al., 2018). Furthermore, realist evaluation has been suggested to enhance RCT design (Bonell et al., 2012). To the authors' knowledge, examples to critique in psychosocial dementia research are scant (Jeon et al., 2019), although combining RCT and realist evaluation as a pragmatic trial has been questioned from a philosophical perspective (see Van Belle et al., 2016).

Theory-driven evaluation approaches adhere to most MRC core elements, can be applied in any phase, and have methodological and reporting standards (Wong et al., 2017). Importantly, these approaches do not claim to offer silver bullets for success. Rather, theory-driven evaluation acknowledges that nothing works everywhere, for everyone, all the time, and according to pragmatic principles (epistemological, methodological, and operational practicality) to develop, test, and refine context-sensitive evidence for more accountable decision-making.

## Toward advancing the field: the METHODEM project

To advance the field of psychosocial dementia research, it is essential to not just discuss exemplary approaches but aim to:

(i) provide a comprehensive overview of which (novel) designs and methodologies are being used;
(ii) reach a consensus on which designs and methodologies (a) integrate the core elements of the MRC framework and (b) suit the objectives of each phase in this area best (i.e., which design/methodology is suitable when, how, and why).

These aims will be targeted in the METHODEM project through a systematic review of the literature covering the past 25 years, and a Delphi study integrating input from researchers, health and social care professionals, policy makers, people living with dementia, and unpaid carers. Gathering, discussing, and disseminating evidence on current research practices and future directions for methodology in psychosocial intervention dementia research has global relevance (WHO, 2017) and may inform further iterations of the MRC framework.

## Conclusions

This article has argued against waste in research endeavors so funding bodies, decision makers, and researchers can consider appropriate designs and methodologies for psychosocial intervention in dementia. We highlight important methodological concerns which should be addressed. To reduce the gap between research and practice and ultimately improve the lives of people living with dementia and unpaid carers, researchers are urged to continue to critically reflect on limitations of currently used methodologies and designs. Guided by the MRC framework, research should consider context and complexity to achieve sustainable impact on the real world and relevance through engagement of people living with dementia, unpaid carers, and other stakeholders.
